# The Scattering Effect-Based Smartphone-Assisted Colorimetric Sensing for Alkaline Phosphatase Detection

**DOI:** 10.3390/bios15100650

**Published:** 2025-10-01

**Authors:** Hao Zhang

**Affiliations:** Chongqing Engineering Research Center of Pharmaceutical Sciences, Chongqing Medical and Pharmaceutical College, Chongqing 401331, China; 2110011@cqmpc.edu.cn

**Keywords:** alkaline phosphatase, the scattering effect, smartphone, Cu-GMP coordination polymer, colorimetric assay

## Abstract

A novel, cost-effective, label-free biosensing strategy has been established for real-time quantification of alkaline phosphatase (ALP) activity, integrating the Tyndall effect with smartphone imaging technology. This method utilizes a handheld laser diode to probe the enzyme-triggered in situ assembly of Cu-guanosine monophosphate (Cu-GMP) coordination polymers, which exhibit tunable Tyndall scattering properties. In the absence of ALP, Cu^2+^ ions chelate with GMP to form Cu-GMP coordination polymers, generating an intense Tyndall effect. Conversely, ALP-mediated hydrolysis of GMP disrupts the formation of Cu-GMP coordination polymers, resulting in diminished light scattering. The intensity of the Tyndall effect is directly proportional to the concentration of Cu-GMP coordination polymers, which in turn correlates with ALP activity levels. A comprehensive investigation of experimental parameters was conducted, including pH, incubation temperature, GMP concentration, incubation time, synthesis duration, and CuSO_4_ concentration. Under optimized conditions, the developed smartphone-assisted colorimetric assay enables the detection of ALP activity within the range of 0.375–3.75 U/mL, with a limit of detection of 0.184 U/mL. The application of this method to serum samples yielded recovery rates ranging from 102.6% to 109.0%. In summary, this smartphone-based colorimetric platform offers a portable and versatile approach for instrument-free detection of ALP activity, with potential applications in point-of-care diagnostics and resource-limited settings.

## 1. Introduction

Alkaline phosphatase (ALP; EC 3.1.3.1) is a vital metalloenzyme in phosphate metabolism of living organisms. Under alkaline conditions, it specifically catalyzes the hydrolysis of phosphate monoesters, producing free inorganic phosphate and alcohols [1,2]. At the protein structural level, ALP exists as a homodimer with a metal-binding site. Each protein monomer is coordinated by two zinc ions and one magnesium ion [3]. The ALP isozyme family is generally classified into two categories: one is tissue-nonspecific alkaline phosphatase (TNAP), and the other is tissue-specific alkaline phosphatase, which is distributed in various tissues and cells such as germ cells, intestines, and placenta [4,5]. TNAP is commonly found in the liver, central nervous system, kidney tissues, and on the basolateral membrane of chondrocytes and osteoblasts; it is essential for controlling the crystallization of hydroxyapatite in the natural development of bones and teeth [6]. When the expression of TNAP becomes imbalanced, it may cause abnormal hydroxyapatite deposition, leading to pathological calcification in blood vessels, kidneys, and tumor tissues [6,7]. Serum ALP levels serve as a reliable biomarker for evaluating bone formation and diagnosing osteoporosis. Consequently, the advancement of a rapid, user-friendly, and portable approach for ALP detection is highly significant for disease diagnosis and health status monitoring.

Several conventional methods have been utilized for ALP determination, encompassing spectrophotometry [8], immunoassay [9], magnetic resonance imaging [10], and mass spectrometry [11]. Additionally, novel techniques including fluorescence analysis [12], surface-enhanced Raman scattering [13], electrochemical analysis [14], and chemiluminescence [15] have been introduced in recent years. Nevertheless, these instrumental analytical techniques usually require complex sample preparation, sophisticated and costly equipment, and professional operators. Challenges remain in ALP detection, especially in terms of speed, simplicity, convenience, and cost-effectiveness, particularly for the initial diagnosis of diseases in home healthcare settings and in remote areas. To address these issues, recent research has focused on developing colorimetric nanosensors that are user-friendly, cost-effective, and can be visually evaluated, thereby improving the monitoring of ALP activity. In particular, noble metal nanoparticles such as colloidal gold and silver nanoparticles have drawn more and more attention as colorimetric signal probes [16,17]. However, synthesizing these probes often involves using noble metal precursors and complex purification procedures. Moreover, accurate quantitative analysis still requires advanced instruments. These limitations undoubtedly result in higher economic and environmental costs for ALP detection.

Point-of-care testing (POCT) is a novel diagnostic technology garnering considerable interest for its notable benefits: speed, accuracy, convenience, and cost-effectiveness. The breakthroughs in biosensors, nanotechnology, and microfluidic technology have greatly promoted the technological innovation of POCT, making detection devices miniaturized and portable. Currently, many POCT products with wireless connectivity have been extensively studied, including pH meters [18], personal glucose meters [19], and smartphones [20]. Among these, the widespread adoption of smartphones has provided a significant opportunity for innovation in biosensor technology. Significant progress has been made in integrating smartphones with colorimetric sensors, attributed to advancements in nanotechnology, computing technology, digital innovation, and the development of user-friendly operating systems. For example, an in situ synthesis method was devised by us to create urease@Prussian blue modified filter paper [21]. The addition of urea prompted urease to catalyze urea hydrolysis, resulting in a pH rise and a subsequent color alteration in Prussian blue. Finally, images were collected by the smartphone and processed using Adobe Photoshop to obtain the color data. The limit of detection (LOD) for urea was 0.27 mM using the colorimetric method. The rapid development of colorimetric methods combined with smartphones has brought about a novel way to generate colorimetric signals. The Tyndall effect is a common light scattering phenomenon observed in colloidal suspensions, and it has been utilized to develop a unique colorimetric indicator for portable smartphone-based sensors [22,23].

**Figure 1:**
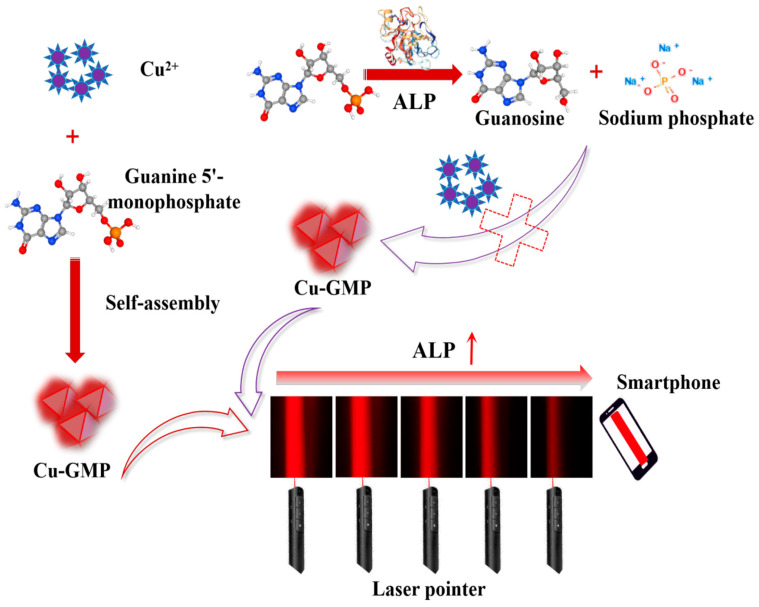
Schematic illustration of the formation of Cu-GMP coordination polymer sheets and the enzyme-controllable in situ formation of Cu-GMP coordination polymer sheets, enabling smartphone-assisted, low-cost, and rapid quantitative detection of alkaline phosphatase based on the Tyndall effect.

In this study, a low-cost, label-free, instrument-free method for quantifying ALP activity using the Tyndall effect with smartphone assistance has been established for the first time. The assay involves analyzing ALP-mediated inhibition of the generation of Cu-guanosine-5′-monophosphate (Cu-GMP) coordination polymer sheets exhibiting the characteristics of the Tyndall effect. In Figure 1, when ALP is absent in the system, Cu^2+^ and GMP form a chelation complex to produce Cu-GMP coordination polymer sheets with the strongest Tyndall effect. However, in the presence of ALP, GMP is hydrolyzed by ALP to generate small molecules like nucleosides and phosphates. These small molecules are unable to form a complex with Cu^2+^, leading to a diminished Tyndall effect. Therefore, the intensity of the Tyndall effect is directly related to the level of Cu-GMP coordination polymer sheets, which in turn is related to the level of ALP activity. By optimizing experimental conditions, including pH, incubation temperature, GMP concentration, incubation time, synthesis time, and CuSO_4_ concentration, the ALP nanosensor designed in this study can detect ALP activity using solely a laser pointer and a smartphone. In addition, the precision of this detection approach was validated through the evaluation of the recovery rate of ALP in serum samples.

## 2. Materials and Methods

### 2.1. Chemicals and Materials

Shanghai Titan Scientific Co., Ltd. (Shanghai, China) supplied guanosine-5′-monophosphate disodium salt hydrate (GMP), potassium chloride, magnesium sulfate (MgSO_4_), and sodium chloride. Alpha-amylase from porcine pancreas, D-lactose, and trypsin from bovine pancreas were bought from Shanghai Yuanye Biological Technology Co., Ltd. (Shanghai, China). Guangdong Guanghua Technology Co., Ltd. (Shantou, China) supplied tris(hydroxymethyl)aminomethane. Shanghai Macklin Biochemical Technology Co., Ltd. (Shanghai, China) supplied collagen, urea, and copper sulfate pentahydrate. Chongqing Chuandong Chemical Co., Ltd. (Chongqing, China) supplied sodium chloride and hydrochloric acid. The normal human serum was bought from Beijing Solarbio Science and Technology Co., Ltd. (Beijing, China).

### 2.2. Instrumentation

A laser pointer (Zhangkun SUPlasericatoy) was purchased from Rui’an LiHao Electronic Technology Co., Ltd. (Rui’an, China). A Huawei Mate 20 Pro smartphone (Huawei Technologies Co., Ltd., (Shenzhen, China)) was used for taking photos. The parameters of the smartphone cameras are set as follows: Professional mode, ISO sensitivity set to 400, shutter speed set to 1/200, exposure compensation set to 0, focusing mode set to continuous focusing, and white balance set to automatic adjustment. The temperature was regulated using a DHG-9035A drying oven (Shanghai Yiheng Scientific Instrument Co., Ltd., Shanghai, China). The SB-4200DT ultrasonic cleaner (Ningbo Scientz Technology Co., Ltd., (Ningbo, China)) was used to prepare solutions. An electronic balance (Sartorius Scientific Instrument (Beijing) Co., Ltd., Beijing, China) was used to weigh reagents. The assessment of surface properties of the material involved the use of a SU8100 Scanning Electron Microscope (Hitachi (Beijing, China), Ltd.) and the acquisition of Transmission Electron Microscopy (TEM) images utilizing a JEM-2100F (JEOL Ltd., (Tokyo, Japan)). A homemade camera obscura was designed, with its specifications detailed in Figure S1.

### 2.3. Preparation of Solutions

A stock solution of CuSO_4_•5H_2_O was prepared by dissolving it in deionized water to achieve a final concentration of 0.66 mM. GMP (0.61 mM), MgSO_4_ (1.0 mM), and ALP (15.0 U/mL) were individually dissolved in Tris-HCl buffer (5.0 mM, pH 7.0). D-lactose, collagen, trypsin, KCl, NaCl, urea, and α-amylase were each prepared at a final concentration of 0.025 mg/mL in Tris-HCl buffer (5.0 mM, pH = 7.0).

### 2.4. Detection of Alkaline Phosphatase by the Smartphone-Assisted Colorimetric Method

In a 2.0 mL sample vial, sequentially add 400.0 µL of GMP solution (0.61 mM), 200.0 µL of MgSO_4_ solution (1.0 mM), and 200.0 µL of ALP solution. For the control group, replace 200.0 µL of ALP solution with 200.0 µL of 5.0 mM Tris-HCl buffer (pH 7.0). Both the sample and control groups are incubated in a DHG-9035A drying oven (Shanghai Yiheng Scientific Instrument Co., Ltd., Shanghai, China) at 50 °C for 20.0 min. Subsequently, 400.0 µL of CuSO_4_•5H_2_O solution is introduced, followed by vortex mixing and a further incubation period of 5.0 min to facilitate Cu-GMP complexation. Following this, the reaction mixture was illuminated using a red laser pointer (Zhangkun SUPlasericatoy, (Hangzhou, China)) to observe the Tyndall effect visually. Finally, to facilitate further quantitative analysis, the images of each Tyndall effect were captured using a Huawei Mate 20 Pro smartphone placed within a self-constructed obscura box to reduce environmental influences. The intensity of the signal, particularly the average gray (AG) value, was assessed using Photoshop CS6 software. The steps for image processing are as follows: First, import the image into Photoshop CS6 software and then crop it in sequence with ratios of 180:30 and 30:30, respectively. Finally, by selecting the gray value mode and analyzing and measuring the cropped image, the AG value can be obtained. The change in the AG (ΔAG) was calculated: ΔAG = AG_blank_ − AG_sample_ (where AG_blank_ and AG_sample_ denote the solution without ALP and the solution containing ALP, respectively).

### 2.5. Selectivity and Interference Study

To determine the reliability of the smartphone-assisted colorimetric method for ALP activity detection, we explored the effects of several common interfering substances present in serum samples. These substances included D-lactose, collagen, trypsin, K^+^, Na^+^, urea, and α-amylase. Selectivity experiments were carried out by detecting interfering substances instead of ALP. The interference experiments were carried out in a similar method, but involved mixing the interfering substances with ALP for analysis. A sample lacking ALP was used as the control group, and the final concentrations of each interfering substance and ALP were set at 0.025 mg/mL and 0.001 mg/mL, respectively.

## 3. Results and Discussion

### 3.1. Characterization of the Cu-GMP

**Figure 2:**
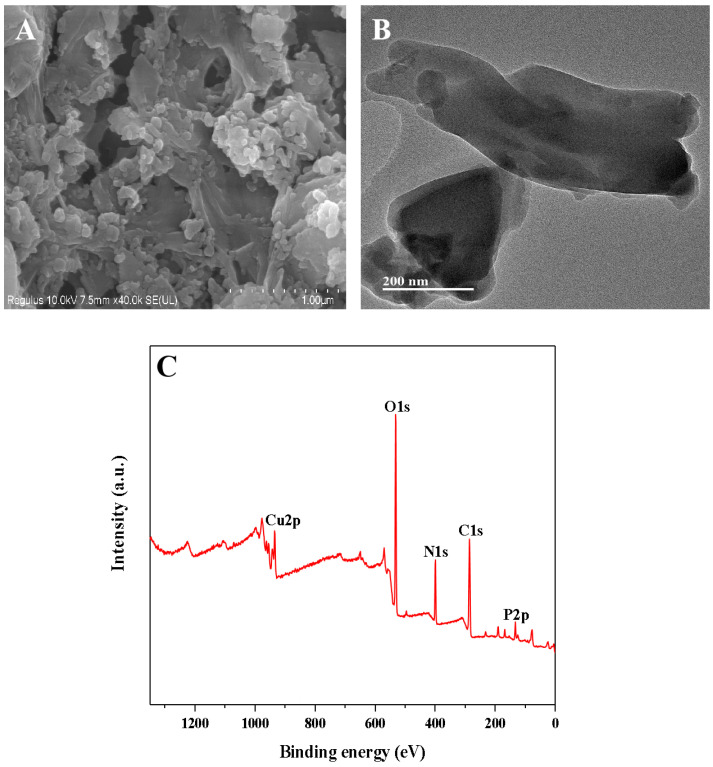
(**A**) SEM and TEM (**B**) images of the Cu-GMP coordination polymer sheets; (**C**) XPS analysis of the Cu-GMP coordination polymer sheets.

**Figure 3:**
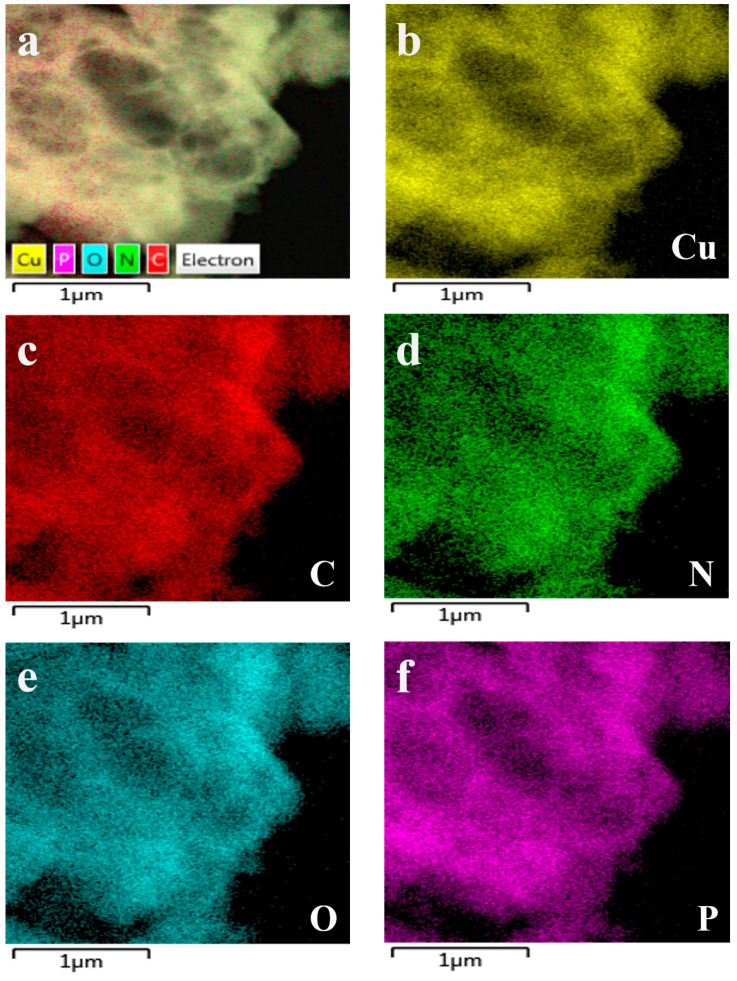
Corresponding elemental mapping images of Cu-GMP coordination polymer sheets. (**a**): element mapping merge; (**b**): Cu element; (**c**): C element; (**d**): N element; (**e**): O element; (**f**): P element.

The Cu-GMP coordination polymer sheets can be synthesized through a coordination reaction between CuSO_4_•5H_2_O and the GMP solution, as depicted in Figure 1. SEM analysis was used to investigate the morphology of the Cu-GMP coordination polymer sheets (Figure 2A). From the SEM images, it is evident that the Cu-GMP coordination polymer sheets have an irregular flaky microstructure, indicating their successful synthesis. Additionally, we employed TEM analysis to further study the microstructures of Cu-GMP. In Figure 2B, the microstructure of Cu-GMP is flaky, further validating the SEM results. The elemental composition of the prepared Cu-GMP coordination polymer sheets was investigated through XPS analysis (Figure 2C). The XPS survey spectra showed signals corresponding to Cu2p, O1s, C1s, N1s, and P2p. The atomic contents of Cu, O, C, N, and P were 3.83%, 32.84%, 44.01%, 15.57%, and 3.75%, respectively. EDS analysis was then used to further confirm the chemical composition of Cu-GMP (Figure 3). EDS mapping images confirmed the successful synthesis of Cu-GMP coordination polymer sheets by detecting carbon, nitrogen, oxygen, phosphorus, and copper on their surfaces. In short, the irregular flaky morphology of Cu-GMP coordination polymers provides a large specific surface area, promoting efficient light scattering through the Tyndall effect. The elemental composition (Cu^2+^ and GMP) contributes to refractive index mismatches between the colloidal particles and the aqueous medium, amplifying the scattering intensity.

### 3.2. Feasibility of Smartphone-Assisted Detection of Alkaline Phosphatase Depending on the Tyndall Effect

**Figure 4:**
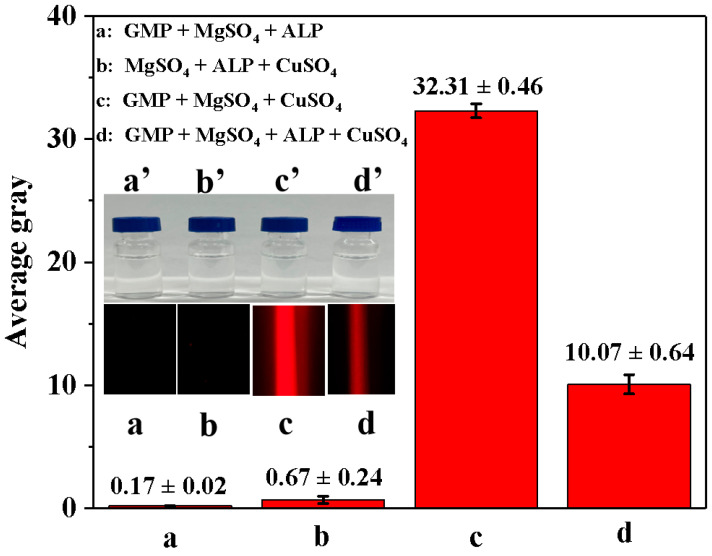
The average gray value of different solutions based on the smartphone-assisted method. (**a**): GMP + MgSO_4_ + ALP mixture solution; (**b**): MgSO_4_ + ALP + CuSO_4_ mixture solution; (**c**): GMP + MgSO_4_ + CuSO_4_ mixture solution; (**d**): GMP + MgSO_4_ + CuSO_4_ + ALP mixture solution. The inset in (**a’**–**d’**) represents the four mixed solutions under sunlight exposure; the inset in (**a**–**d**) represents the four mixed solutions under red laser exposure.

We investigated the feasibility of using enzyme-controllable in situ formation of Cu-GMP coordination polymer sheets for smartphone-assisted colorimetric detection of ALP activity. In Figure 4, all mixed solutions appeared colorless under sunlight (a’–d’). Additionally, when illuminated with a red laser pointer, no distinct colors were observed in the GMP + MgSO_4_ + ALP mixture solution (Figure 4 in a) or the MgSO_4_ + ALP + CuSO_4_ mixture solution (Figure 4 in b), with AG values of 0.17 ± 0.02 and 0.67 ± 0.24, respectively. However, when the GMP + MgSO_4_ + CuSO_4_ mixture solution was illuminated with a laser pointer, a characteristic Tyndall signal was visible (Figure 4 in c), with an AG value of 32.31 ± 0.46, suggesting that the colloidal material was formed from GMP and CuSO_4_. The GMP + MgSO_4_ + CuSO_4_ + ALP mixture solution showed a weakened Tyndall signal when illuminated with a red laser pointer (Figure 4 in d), with an AG value of 10.07 ± 0.64, indicating that ALP might inhibit the formation of Cu-GMP coordination polymer sheets. Moreover, an experimental study was conducted to verify the feasibility of using a smartphone to capture Tyndall effect images and then extract AG values using Adobe Photoshop CS6 for detecting ALP activity. In Figure 4, the strength of the Tyndall effect was related to the AG values, which implies that using a smartphone along with the Tyndall effect allows for quick and convenient detection of ALP activity.

### 3.3. Optimization of the Detection Conditions for ALP Activity

**Figure 5:**
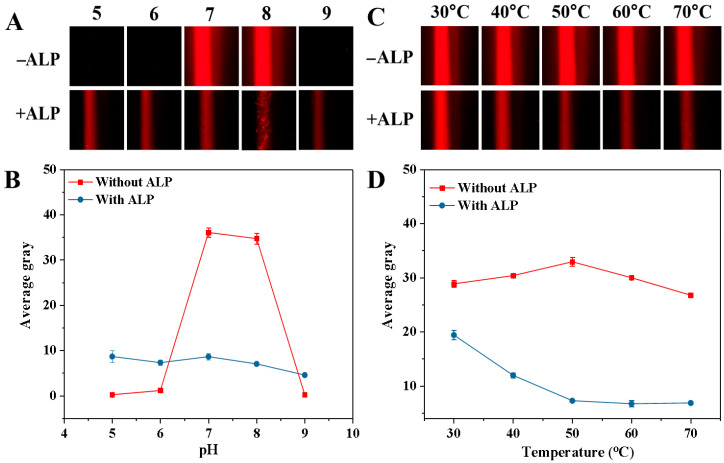
Effects of pH (**A**,**B**) and temperature (**C**,**D**) on ALP-mediated Cu-GMP coordination polymer formation in the presence or absence of ALP. (**A**): Tyndall images across pH = 5.0–9.0; (**B**): Quantitative analysis of the average gray value of the Tyndall images in (**A**) (*n* = 3, error bars = standard deviation, SD); (**C**): Tyndall images across incubation temperature from 30 °C to 70 °C; (**D**): Quantitative analysis of the average gray value of the Tyndall images in (**C**) (*n* = 3, error bars = standard deviation, SD).

To achieve the optimal performance in detecting ALP activity, we studied how experimental parameters such as pH, incubation temperature, GMP concentration, incubation time, synthesis time, and CuSO_4_ concentration influenced AG values. The pH of the buffer could affect both the Tyndall effect of the blank sample and ALP activity. Thus, the impact of the 5.0 mM Tris-HCl buffer solution with a pH range from 5.0 to 9.0 on the AG value was examined, and the corresponding Tyndall effect picture and results are shown in Figure 5A and B, respectively. When ALP was not present, the AG value rose from pH 5.0 to 7.0 and then gradually decreased from pH 7.0 to 9.0 (Figure 5B). Notably, at pH 5.0, 6.0, and 9.0, the AG values were close to zero. However, at pH 7.0 and 8.0, the AG values were both relatively high (The relevant Tyndall images are also shown in Figure 5A). This could be because GMP is more likely to chelate with Cu^2+^ to form a colloidal solution under neutral and weakly alkaline conditions. But when ALP was present in the mixed solution, the AG value tended to stay stable (The relevant Tyndall images are also shown in Figure 5A). This might be due to ALP catalyzing the hydrolysis of GMP, thereby hindering the formation of the Cu-GMP coordination polymer sheets. To achieve the optimal sensitivity in detecting ALP, the change in AG (ΔAG) was calculated: ΔAG = AG_blank_ − AG_sample_ (where AG_blank_ and AG_sample_ represent the AG values of the solution without ALP and the solution containing ALP, respectively). In Figure 5B, the most notable change in ΔAG value occurs at pH 7.0. Thus, pH 7.0 was chosen for the next study. The temperature of the reaction system significantly regulated the catalytic activity of ALP and the formation process of Cu-GMP colloids. Hence, we investigated the effect of temperature on AG value, and the corresponding Tyndall effect images are shown in Figure 5C. In Figure 5D, without ALP, the AG value gradually increased from 30 °C to 50 °C and then gradually decreased as the temperature exceeded 50 °C, indicating that temperature impacts the formation of Cu-GMP colloids. Conversely, when ALP was present, the AG value gradually decreased from 30 °C to 50 °C and then remained stable when the temperature was above 50 °C. This might be attributed to the enhanced ALP activity at high temperatures, which reduces the formation of Cu-GMP colloids. However, the most notable change in ΔAG value occurred at 50 °C. Thus, 50 °C was chosen as the reaction temperature.

GMP can not only chelate with Cu^2+^ to form a Cu-GMP colloidal solution, but it can also be hydrolyzed by ALP. So, we analyzed how different GMP concentrations affected AG value. The Tyndall effect images and results were depicted in Figure S2A and B, respectively. Without ALP, AG values increased gradually as the GMP concentration rose from 0.31 mM to 1.23 mM. Once the GMP concentration surpassed 1.23 mM, AG values stabilized. This was probably because as the GMP concentration increased, more Cu-GMP colloids were formed. But when there was too much GMP in the solution, further increasing its amount did not lead to more Cu-GMP colloids, causing AG values to stabilize. On the other hand, with or without ALP, the change in AG values were consistent. This was due to the presence of ALP, which led to a reduction in the generated Cu-GMP colloids. Nevertheless, as the GMP concentration increased, excess GMP chelated with Cu^2+^, causing AG values to rise until they stabilized. The optimal GMP concentration was determined to be 0.61 mM, as this concentration led to the maximum increment in AG value. The effect of incubation time between ALP and GMP on the Tyndall effect was shown in Figure S2C,D. Figure S2D showed how AG values changed with incubation time. Without ALP, AG values remained almost the same as the incubation time increased. However, with ALP present, AG values decreased gradually with increasing incubation time and became nearly constant after 30.0 min, and the corresponding Tyndall effect picture was shown in Figure S2C. The optimal incubation time was determined to be 20.0 min, as this was when the maximum increment of AG value occurred.

Additionally, the effect of reaction time between CuSO_4_ and GMP on the Tyndall effect is depicted in Figure S3A,B. Figure S3B illustrates the variation in AG values with the reaction time between CuSO_4_ and GMP. Without ALP added, AG values increased as the reaction time increased from 1.0 min to 5.0 min. However, when the reaction time exceeded 5.0 min, AG values tended to stabilize, with the corresponding Tyndall effect images shown in Figure S3A. This was likely because as the reaction time increased, more Cu-GMP colloids were produced. However, with further extension of the reaction time, the amount of generated Cu-GMP colloids did not increase, resulting in stable AG values. On the other hand, the change in AG values remained nearly constant as the reaction time increased in the presence of ALP. The optimal reaction time occurred at 5.0 min, as this specific time point led to the maximum increment in AG value. Finally, we studied the effects of CuSO_4_ concentration on the Tyndall effect (Figure S3C,D). In Figure S3D, without ALP in the system, AG values increased as the CuSO_4_ concentration rose from 0.33 mM to 0.66 mM and then remained almost unchanged. However, with ALP present, AG values barely changed as the CuSO_4_ concentration increased, with the corresponding Tyndall effect images shown in Figure S3C. Since the maximum increment in AG value occurred at 0.66 mM, this concentration was chosen as the optimal CuSO_4_ concentration for subsequent experiments.

### 3.4. Analytical Performance

**Figure 6:**
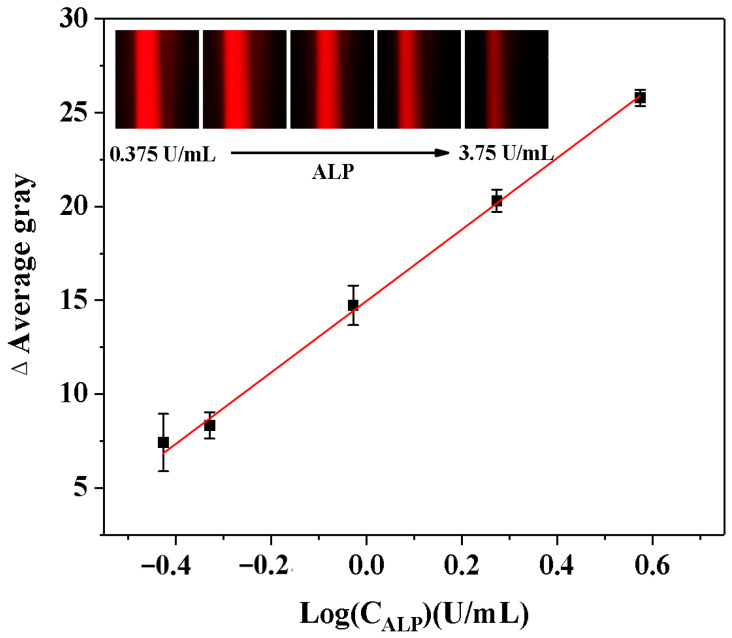
Calibration curves describing the relationships between the AG change (ΔAG) values of the Tyndall effect results and the logarithm values of the ALP activity (LogC_ALP_). (Insets: the corresponding Tyndall effect images with different activities of ALP).

The relative standard deviation (RSD%) of AG values was calculated to evaluate the repeatability of this method in detecting ALP activity. The developed ALP analysis demonstrated reliable reproducibility and accuracy, as evidenced by an RSD% value of 2.3% from five consecutive measurements. After that, ALP samples with different levels (0.375–3.75 U/mL) were analyzed to evaluate the analytical performance of the smartphone-assisted ALP detection method depending on the Tyndall effect. As demonstrated in Figure 6, the Tyndall effect was significantly weakened with increasing ALP activity. The logarithm of ALP activity demonstrated a robust linear relationship with the ΔAG value of the solution. Thus, ALP detection was reliable within the range of 0.375 to 3.75 U/mL, with a LOD of 0.184 U/mL (LOD = 3*δ/slope, n = 11). This linear relationship could be expressed by the regression equation: ΔAG = 19.057LogC_ALP_ (U/mL) + 14.928 (R^2^ = 0.9989). Our LOD falls within the early pathological range of ALP, enabling the detection of subtle ALP elevations that precede severe symptoms. Moreover, this study describes the technique for evaluating the analytical performance in comparison with alternative ALP activity analysis (Table 1). The novelty of this approach lies in its simplification of the complex traditional detection process (with an operation time of only 25.0 min), without the need for large-scale equipment (with relatively low cost) and avoidance of biomolecule labeling steps.

### 3.5. Selectivity and Interference Study

**Figure 7:**
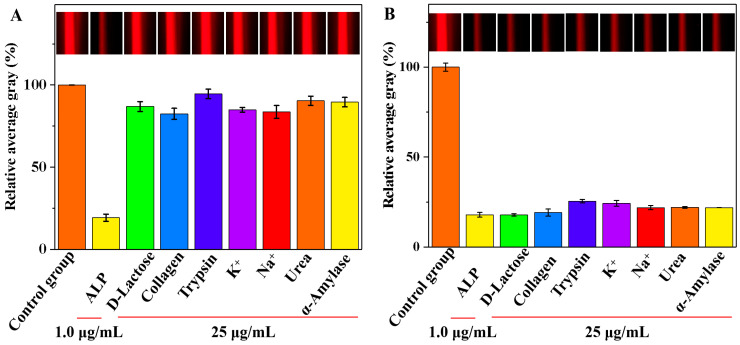
The selectivity (**A**) and interference study (**B**) of the developed method for the detection of ALP activity (Insets: the corresponding images).

Selectivity and interference analyses were conducted to determine the specificity of the smartphone-assisted colorimetric method for the detection of ALP in complex serum matrices. Under optimal conditions, we recorded the relative AG values of various substances that might be present in actual samples, including D-lactose, collagen, trypsin, K^+^, Na^+^, urea, and α-amylase. A solution without ALP added was selected as the control group. In Figure 7A, the presence of ALP in the system had the most significant effect on the relative AG value compared with that of the control group. In contrast, the impact of other interfering substances on the relative AG values were negligible. Additionally, the Tyndall effect images remained unchanged in the presence of interfering substances compared with those of the control group (insert in Figure 7A). The results suggested that the smartphone-assisted colorimetric method is highly effective in selectively detecting ALP activity. As shown in Table 1, the selectivity of each method was evaluated by assessing the relative concentrations and interference rate of interfering substances and the target analyte. The results showed that this study exhibited selectivity comparable to that of published methods. The interference study was further explored, and trypsin and K^+^ had a slight effect on the relative AG values (Figure 7B). The other interfering substances had minimal impacts on the relative AG values, which emphasizes the high accuracy of the designed method for the detection of ALP activity. Moreover, the Tyndall effect images were not affected by the presence of interfering substances when compared with those of the control group (insert in Figure 7B).

### 3.6. Real Sample Analysis

To evaluate the analytical performance of the proposed smartphone-assisted colorimetric method in real samples, a serum sample was chosen and spiked with ALP at different activities. Prior to conducting subsequent analysis experiments, the serum samples were diluted 500 times. In Table 2, it can be observed that the addition of ALP in serum samples at three varying activities (0.5, 1.0, and 2.0 U/mL) yielded recoveries ranging from 102.6% to 109.0%. The results clearly demonstrate that the developed assay can be used for the spiked determination of ALP activity in real samples.

## 4. Conclusions

In this study, a straightforward, cost-effective, and widely applicable colorimetric assay was developed. This method integrates the coordination characteristics of Cu-GMP coordination polymer sheets and the Tyndall effect, and utilizes a smartphone to provide a simple and cost-effective method for the rapid and accurate determination of ALP activity. The method developed in this study only requires simple mixed chelating reagents, without the need to prepare special nano-probes. A key advantage of this method is that it only requires a laser pointer and a smartphone to conduct cost-effective, portable analysis of ALP activity, eliminating the need for complex instruments. Based on the aforementioned advantages, this method is tailored for scenarios characterized by limited resources and on-site operations. Its primary application fields encompass: (1) primary healthcare clinics in remote areas; (2) household health monitoring; (3) on-site emergency diagnostics. Although this method cannot replace highly sensitive laboratory techniques for ultra-low concentration detection, it provides a convenient and cost-effective approach for early disease detection in scenarios where traditional ALP detection methods are unavailable. The main focus of this study was to detect a pathological increase in ALP, which is the primary objective for conducting POCT in resource-limited settings. In summary, the smartphone-assisted Tyndall effect assay shows great potential as a signal readout method for POCT applications. It particularly enables rapid, user-friendly, and highly sensitive detection of ALP activity, making it an ideal choice for implementation in resource-limited settings.

## Figures and Tables

**Table 1:** Comparisons of the analytical methods for the detection of ALP.

Materials	Equipment/Consumable Requirements	Total Operation Time	Detection Methods	Linear Range (U/mL)	Sensitivity (LOD)	Selectivity	Ref.
Paper-based immunosensor	Smartphone and immunomodified test strips	>6.5 h	Colorimetric analysis with smartphone	10.0–1000.0	0.87 U/mL	/	[24]
Eu/Dibenzoyl methane@SiO_2_@SiO_2_	UV–Vis spectrophotometer	>27.0 h	UV–Vis	0.02–0.09	0.0072 U/mL	Moderate	[25]
3D DNA and paper device	Smartphone and test strips	>16.0 h	Colorimetric analysis with smartphone	0–0.8	0.0134 U/mL	/	[26]
Au nanoparticles	Smartphone and gold nanoparticles	>0.5 h	Colorimetric analysis	0.025–0.25	0.02757 U/mL	Moderate	[27]
Coumarin@Tb-GMP nanoparticles	Fluorescence spectrophotometer	>1.0 h	Fluorescent analysis	0.025–0.2	0.01 U/mL	High	[28]
Amifostine	Glucose meter and matching test strips	5.0 min	Personal glucose meter	330−3330	130 U/mL	High	[29]
Cu-GMP coordination polymer sheets	Laser pointer, smartphone and homemade camera obscura	25.0 min	Colorimetric analysis with smartphone	0.375–3.75	0.184 U/mL	High	This study

“/”: The concentrations of the interfering substances and the target analytes were not provided.

**Table 2:** Recovery study of ALP in serum sample (*n* = 3).

Sample	Added (U/mL)	Found ± SD (U/mL)	Recovery (%)
Human serum	0	0	-
	0.5	0.51 ± 0.04	102.6
	1.0	1.08 ± 0.11	107.7
	2.0	2.18 ± 0.12	109.0

## Data Availability

The original contributions presented in this study are included in the article/Supplementary Material. Further inquiries can be directed to the corresponding author.

## Supplementary Materials

The following supporting information can be downloaded at: https://www.mdpi.com/article/10.3390/bios15100650/s1, Figure S1. The structure and specification of a homemade camera obscura. Figure S2. Effects of GMP concentration (A,B) and incubation time (C,D) on ALP-mediated Cu-GMP coordination polymer formation in the presence or absence of ALP. A: Tyndall images across GMP concentration from 0.31 mM to 2.46 mM; B: Quantitative analysis of the average gray value of the Tyndall images in A (*n* = 3, error bars = standard deviation, SD); C: Tyndall images across incubation time from 5.0 min to 50.0 min; D: Quantitative analysis of the average gray value of the Tyndall images in C (*n* = 3, error bars = standard deviation, SD). Figure S3. Effects of synthesis time (A,B) and CuSO_4_ concentration (C,D) on ALP-mediated Cu-GMP coordination polymer formation in the presence or absence of ALP. A: Tyndall images across synthesis time from 1.0 min to 20.0 min; B: Quantitative analysis of the average gray value of the Tyndall images in A (*n* = 3, error bars = standard deviation, SD); C: Tyndall images across CuSO_4_ concentration from 0.33 mM to 2.67 mM; D: Quantitative analysis of the average gray value of the Tyndall images in C (*n* = 3, error bars = standard deviation, SD).

## Abbreviations

The following abbreviations are used in this manuscript:

ALP	Alkaline phosphatase
GMP	Guanosine-5′-monophosphate
TNAP	Tissue-nonspecific alkaline phosphatase
POCT	Point-of-care testing
MgSO_4_	Magnesium sulfate
AG	Average gray
LOD	Limit of detection
