# Supplementary material for: The Scattering Effect-Based Smartphone-Assisted Colorimetric Sensing for Alkaline Phosphatase Detection
Source: Biosensors (Basel). 2025 Oct 1;15(10):650. doi: 10.3390/bios15100650 (PMC12563470; doi:10.3390/bios15100650)
Supplement: Supplementary file 1 [file biosensors-15-00650-s001.zip › biosensors-3812071-supplementary.pdf]

biosensors

Supplementary Material

The Scattering Effect-Based Smartphone-Assisted Colorimetric Sensing for Alkaline Phosphatase Detection

Hao Zhang*

Chongqing Engineering Research Center of Pharmaceutical Sciences, Chongqing Medical and Pharmaceutical College, Chongqing 401331, China
* Correspondence: 2110011@cqmpc.edu.cn

Table of contents

Figure S1. The structure and specification of a homemade camera obscura.

Figure S2. Effects of GMP concentration (**A, B**) and incubation time (**C, D**) on ALP-mediated Cu-GMP coordination polymer formation in the presence or absence of ALP. **A**: Tyndall images across GMP concentration from 0.31 mM to 2.46 mM; **B**: Quantitative analysis of the average gray value of the Tyndall images in **A** (n = 3, error bars = standard deviation, SD); **C**: Tyndall images across incubation time from 5.0 min to 50.0 min; **D**: Quantitative analysis of the average gray value of the Tyndall images in C (n = 3, error bars = standard deviation, SD).

Figure S3. Effects of synthesis time (**A, B**) and $CuSO_4$ concentration (**C, D**) on ALP-mediated Cu-GMP coordination polymer formation in the presence or absence of ALP. **A**: Tyndall images across synthesis time from 1.0 min to 20.0 min; **B**: Quantitative analysis of the average gray value of the Tyndall images in **A** (n = 3, error bars = standard deviation, SD); **C**: Tyndall images across $CuSO_4$ concentration from 0.33 mM to 2.67 mM; **D**: Quantitative analysis of the average gray value of the Tyndall images in C (n = 3, error bars = standard deviation, SD).

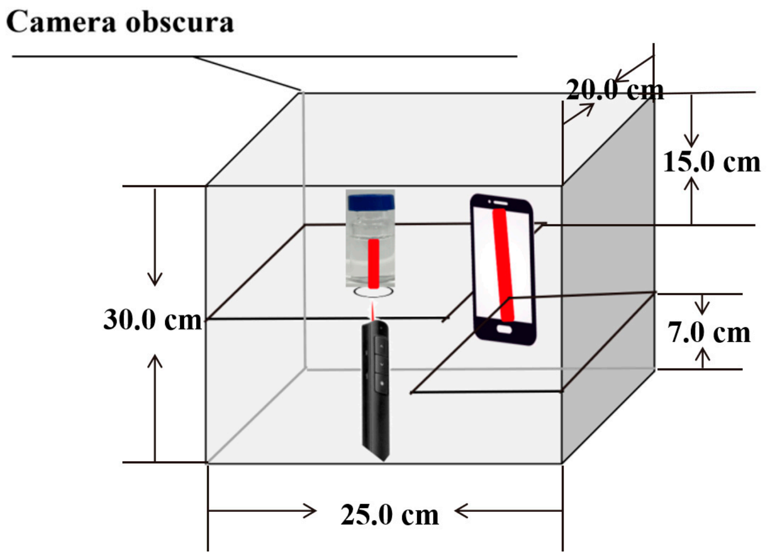

Figure S1. The structure and specification of a homemade camera obscura.

 https://doi.org/10.3390/xxxxx

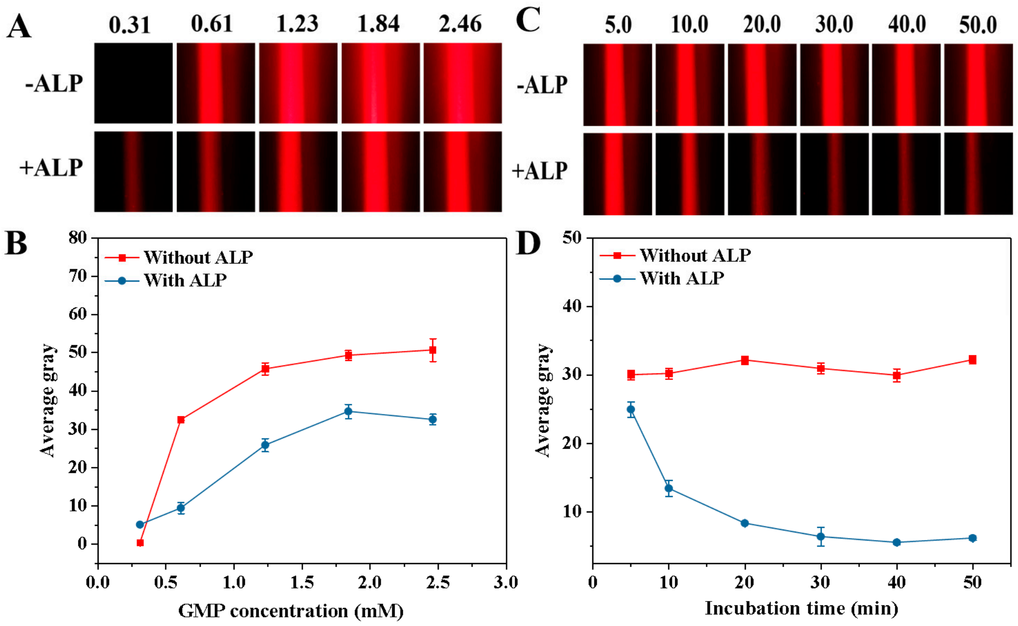

Figure S2. Effects of GMP concentration (**A, B**) and incubation time (**C, D**) on ALP-mediated Cu-GMP coordination polymer formation in the presence or absence of ALP. **A**: Tyndall images across GMP concentration from 0.31 mM to 2.46 mM; **B**: Quantitative analysis of the average gray value of the Tyndall images in **A** (*n* = 3, error bars = standard deviation, SD); **C**: Tyndall images across incubation time from 5.0 min to 50.0 min; **D**: Quantitative analysis of the average gray value of the Tyndall images in C (*n* = 3, error bars = standard deviation, SD).

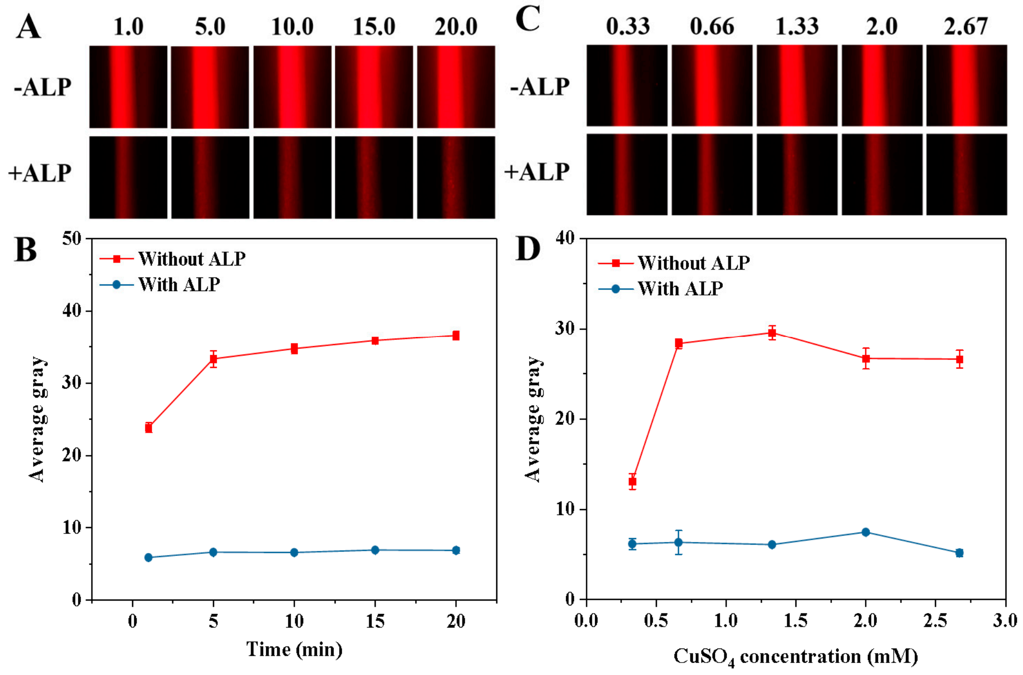

Figure S3. Effects of synthesis time (**A, B**) and $CuSO_4$ concentration (**C, D**) on ALP-mediated Cu-GMP coordination polymer formation in the presence or absence of ALP. **A**: Tyndall images across synthesis time from 1.0 min to 20.0 min; **B**: Quantitative analysis of the average gray value of the Tyndall images in **A** (*n* = 3, error bars = standard deviation, SD); **C**: Tyndall images across $CuSO_4$ concentration from 0.33 mM to 2.67 mM; **D**: Quantitative analysis of the average gray value of the Tyndall images in C (*n* = 3, error bars = standard deviation, SD).
